# A perfusion bioreactor-based 3D model of the subarachnoid space based on a meningeal tissue construct

**DOI:** 10.1186/s12987-019-0137-6

**Published:** 2019-06-13

**Authors:** Albert Neutzner, Laura Power, Markus Dürrenberger, Hendrik P. N. Scholl, Peter Meyer, Hanspeter E. Killer, David Wendt, Corina Kohler

**Affiliations:** 1 Department of Biomedicine, University Hospital Basel & University Basel, Hebelstr. 20, 4031 Basel, Switzerland; 2 Department of Ophthalmology, University Hospital Basel & University Basel, Hebelstr. 20, 4031 Basel, Switzerland; 3 Department of Surgery, University Hospital Basel & University Basel, Hebelstr. 20, 4031 Basel, Switzerland; 4 Department of Biomedical Engineering, University Hospital Basel & University Basel, Hebelstr. 20, 4031 Basel, Switzerland; 5 Swiss Nanoscience Institute, University Basel, Klingelbergstr. 50, 4056 Basel, Switzerland; 6 Department of Ophthalmology, University Hospital Basel & University Basel, Mittlere Str. 91, 4056 Basel, Switzerland; 7 Institute of Molecular and Clinical Ophthalmology, Mittlere Str. 91, 4056 Basel, Switzerland; 8 Department of Ophthalmology, Kantonsspital Aarau, Tellstrasse 25, 5001 Aarau, Switzerland

**Keywords:** Optic nerve, Subarachnoid space, Meningothelial cells, Bioreactor, Optic nerve compartment syndrome, Cerebrospinal fluid

## Abstract

**Background:**

Altered flow of cerebrospinal fluid (CSF) within the subarachnoid space (SAS) is connected to brain, but also optic nerve degenerative diseases. To overcome the lack of suitable in vitro models that faithfully recapitulate the intricate three-dimensional architecture, complex cellular interactions, and fluid dynamics within the SAS, we have developed a perfusion bioreactor-based 3D in vitro model using primary human meningothelial cells (MECs) to generate meningeal tissue constructs. We ultimately employed this model to evaluate the impact of impaired CSF flow as evidenced during optic nerve compartment syndrome on the transcriptomic landscape of MECs.

**Methods:**

Primary human meningothelial cells (phMECs) were seeded and cultured on collagen scaffolds in a perfusion bioreactor to generate engineered meningeal tissue constructs. Engineered constructs were compared to human SAS and assessed for specific cell–cell interaction markers as well as for extracellular matrix proteins found in human meninges. Using the established model, meningeal tissue constructs were exposed to physiological and pathophysiological flow conditions simulating the impaired CSF flow associated with optic nerve compartment syndrome and RNA sequencing was performed.

**Results:**

Engineered constructs displayed similar microarchitecture compared to human SAS with regards to pore size, geometry as well as interconnectivity. They stained positively for specific cell–cell interaction markers indicative of a functional meningeal tissue, as well as extracellular matrix proteins found in human meninges. Analysis by RNA sequencing revealed altered expression of genes associated with extracellular matrix remodeling, endo-lysosomal processing, and mitochondrial energy metabolism under pathophysiological flow conditions.

**Conclusions:**

Alterations of these biological processes may not only interfere with critical MEC functions impacting CSF and hence optic nerve homeostasis, but may likely alter SAS structure, thereby further impeding cerebrospinal fluid flow. Future studies based on the established 3D model will lead to new insights into the role of MECs in the pathogenesis of optic nerve but also brain degenerative diseases.

**Electronic supplementary material:**

The online version of this article (10.1186/s12987-019-0137-6) contains supplementary material, which is available to authorized users.

## Background

The flow of CSF within the SAS is indispensable for maintaining brain, spinal cord but also optic nerve function. CSF not only provides important cushioning, but is also essential for central nervous system homeostasis, sustaining nutrient supply to neuronal-glial networks, mediating transportation of signaling molecules and removing toxic metabolites [1]. Changes in CSF pressure, flow dynamics and composition have been related to age-linked and neurodegenerative diseases [2–4].

The SAS imposes a unique environment to its cellular component, the meningothelial cells (MECs) which are a key constituent of the dura mater and the leptomeninges (arachnoid and pia mater). The outermost meningothelial layer of the arachnoid fulfills a crucial function, forming a tight junction barrier thereby separating the CSF-filled SAS from the circulatory system in the dura [5]. From this layer, MECs extend into the SAS covering the collagen-enforced trabeculae, pillars and septae which ultimately submerge into the pia. As part of the pia, MECs form a thin mono-layer that is connected by gap-junctions and is in direct proximity to the basement membrane [6].

Within this intricate environment MECs are being subjected and adapted to continuous CSF flow. Under physiological conditions, CSF flow is tightly regulated and is likely important for maintaining MEC morphological properties as well as their physiological role. MECs synthesize key extracellular matrix (ECM) proteins of the meninges such as various collagens, as well as fibronectin, laminin and tenascin, important for pia basal lamina integrity and thus for neuronal support and function [7]. MECs also play a central role in CSF conditioning by secreting immune-modulating mediators including cytokines and chemokines, as well as neurotrophic factors and retinoic acid, which have been shown to promote axonal regeneration and differentiation [8–11]. In addition, MECs have been implicated in the clearance of waste products from the SAS through phagocytosis which is important for maintenance of neuronal tissue homeostasis [9, 12]. Taken together, alterations in the microenvironment of MECs could not only have potentially deleterious effects on MECs, but ultimately as well on neuronal function and survival.

To date, research on MECs has been hampered by the lack of suitable in vitro models that are capable of recapitulating the intricate three-dimensional architecture, complex cell–cell and cell–matrix interactions, and fluid dynamics within the SAS. Therefore, in this work we aimed to generate a state-of-the-art perfusion bioreactor-based 3D model of the SAS, based on an in vitro engineered meningeal tissue construct.

A pathology that highlights the importance of continuous CSF flow is optic nerve compartment syndrome (ONCS). ONCS is characterized by stagnating CSF flow within the peri-optic nerve SAS and is linked to a variety of optic neuropathies, such as normal tension glaucoma, papilledema and visual impairment and intracranial pressure syndrome [13–15]. Using the established model system, we finally aimed to simulate conditions of optic nerve compartment syndrome to study the effects of pathophysiological flow conditions on MEC function. Performing RNA sequencing, gene ontology, as well as pathway/network analyses, we determined that under pathological flow conditions genes involved in major physiological MEC functions were differentially regulated as compared to physiological flow conditions, thereby providing novel clues for a role of these cells in the pathogenesis of ONCS.

## Methods

### Perfusion bioreactor-based in vitro model of the subarachnoid space

#### Model establishment

**Fig. 1:**
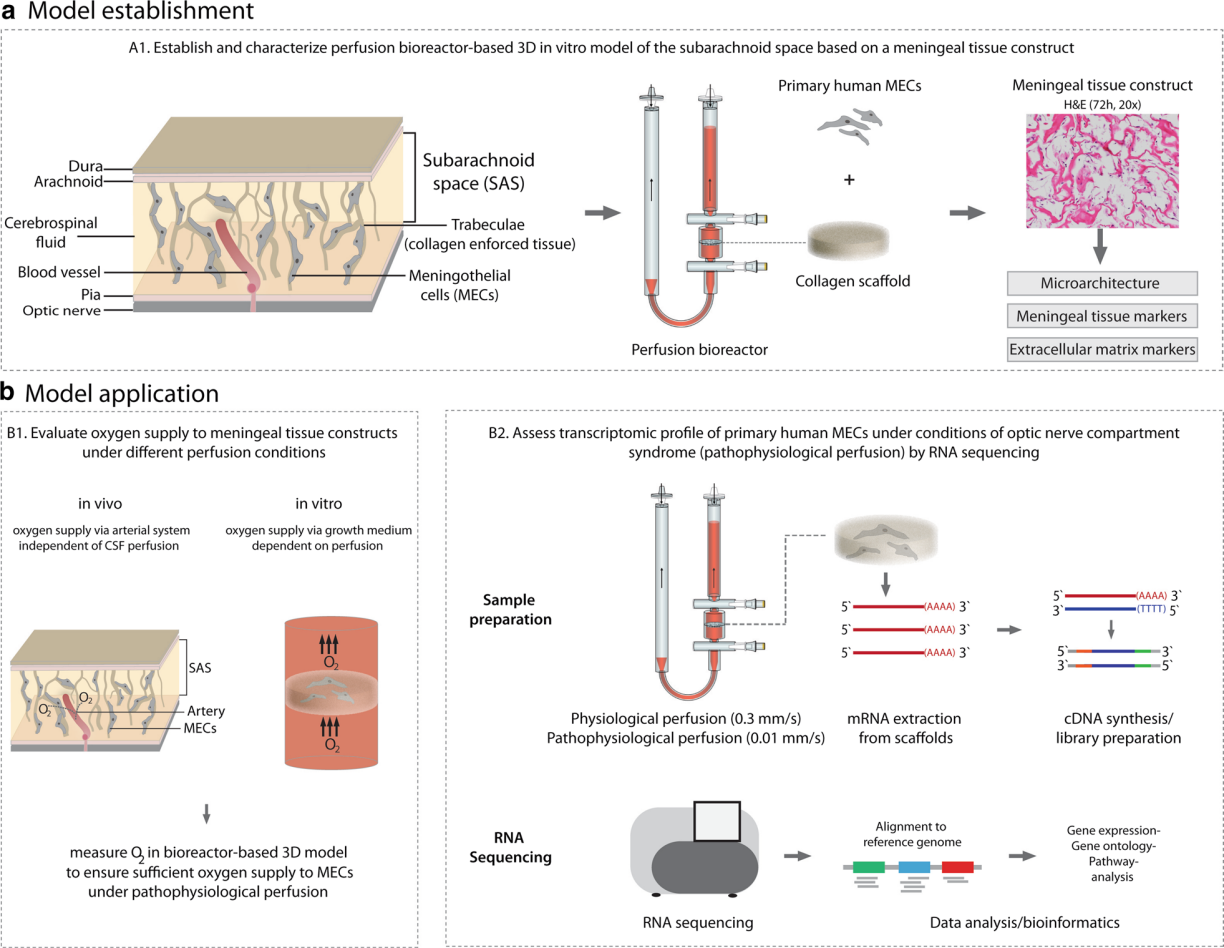
Overview of model establishment and application. **a** Model establishment. To mimic SAS morphology and composition, U-CUP perfusion bioreactor was employed together with a collagen scaffold and phMECs were perfusion seeded at 1.0 mm/s for 24 h and then cultivated for 72 h at a superficial velocity of 0.3 mm/s, to achieve a meningeal tissue construct. Tissue construct was characterized by assessing microarchitectural similarity between in vivo SAS and the engineered meningeal tissue construct employing scanning electron microscopy and by staining for meningeal tissue and extracellular matrix markers using IHC. **b** Model application. B1 To ensure sufficient oxygen supply to phMECs during pathophysiological perfusion, O_2_ saturation was measured during pathophysiological perfusion conditions. B2 Transcriptomic profile of phMECs was assessed during physiological and pathophysiological perfusion conditions of ONCS using RNA sequencing

A tissue engineering strategy was used to establish a 3D in vitro model of the subarachnoid space based on a meningeal tissue construct. To this end, primary human meningothelial cells (phMECs) of leptomeningeal origin (Sciencell; Cat.no. #1400; USA) were seeded at passage 6/7 and cultured in a porous collagen scaffold (ULTRAFOAM™; Bard, USA) within a perfusion bioreactor (Cellec Biotek, Switzerland) (Fig. 1A1).

The perfusion bioreactor system was used to first perfuse a cell suspension (1E + 06 phMECs) directly through the pores of the 3D scaffold (8 mm diameter × 2 mm height) at a superficial velocity of 1.0 mm/s, in order to seed cells uniformly throughout the scaffold volume. After 24 h of perfusion seeding, the flowrate was reduced, and phMECs were cultured under perfusion (superficial velocity of 0.3 mm/s) for an additional 72 h to engineer a meningeal tissue construct.

#### Morphological characterization of meningeal tissue constructs

To assess the microarchitectural similarity between the engineered meningeal tissue constructs and in vivo SAS, scanning electron microscopy of an empty collagen scaffold, a meningeal tissue construct as well as of the SAS of a human optic nerve section (mid-orbital) was performed. To this end, human optic nerve was removed postmortem from a healthy donor, within 7 h after death. Written informed consent was obtained as part of the agreement for autopsy. Both globe and optic nerve was fixed in 2% glutaraldehyde (0.1 M cacodylate buffer) for 1 week. After dehydration of optic nerve segment using an acetone series, critical point drying was performed. Samples were mounted onto aluminium holders and sputtered with gold (15 nm). Analyses was done on an SEM 505 (Philips, Einthoven, Netherlands). Meningeal tissue constructs were created as stated in the previous section. After 72 h of culturing constructs were removed from the bioreactor culture chamber, washed in TAM buffer for 10 s, ultra rapid frozen in propane (according to Dubochet), transferred to liquid nitrogen and broken into pieces using a scalpel. This was followed by a 12 h freeze-drying program (stepwise increasing the temperature from 172 Kelvin to room temperature) using an EMS775X freeze drier (Quorum, UK). Samples were mounted onto aluminium holders and sputtered with gold (20 nm) at a vacuum of 2 × 10^−5^ mbar. Empty scaffold and meningeal tissue construct were analyzed using a Nova Nano SEM 230 (FEI, NL).

Meningeal tissue constructs were characterized evaluating cell–cell as well as ECM protein expression, both characteristic features of meningeal tissue. Following 72 h of perfusion culture, engineered constructs were harvested, formalin fixed, paraffin embedded, and cross-sectioned (4 μM thick slices). To evaluate the cell distribution, hematoxylin and eosin (H&E) staining was performed. For evaluation of cell–cell interaction markers scaffolds were stained using the following antibodies: Junctional adhesion molecule A, (Novus Biologicals, H00050848-M01), occludin (abcam, #ab31721), claudin 5 (abcam, #ab15106), connexin 43 (Sigma, #C6219), connexin 26 (abcam, #ab38584), desmoplakin I + II (Progen, #65146). For assessment of ECM markers, engineered constructs were stained using the following antibodies: Pro-collagen I (Fitzgerald, #10R-1396), collagen II (MP BIOMEDICALS, #0863171), IV (Ventana, #760-2632), laminin (Thermo, #RB-082-A) and tenascin (FMI) (Fig. 1A1).

### Perfusion bioreactor-based in vitro model of ONCS

#### Simulation of ONCS

Using our bioreactor-based model of the SAS, phMECs were next cultured under pathophysiological perfusion conditions to simulate ONCS. phMEC-based constructs were engineered as described above. Flow rates were selected based on diffusion MRI measurements of flow-range ratios between the intracranial cavity and the subarachnoid space of the optic nerves (our unpublished observations). Regarding pathophysiological CSF flow occurring during ONCS, we used a flowrate in the bioreactor that was dramatically reduced to 2.5% of the normal flow, allowing for significantly inhibited flow while at the same time maintaining sufficient mass transport of oxygen to cells in the 3D construct, preventing hypoxic conditions. After 72 h of culture, flow rates for bioreactors simulating pathophysiological perfusion conditions were reduced to 0.01 mm/s for 24 h (n = 6). As controls, engineered constructs were also maintained at physiological flow rates of 0.3 mm/s for 24 h (n = 6) (Fig. 1B2). Oxygen measurements were performed to monitor oxygen concentrations supplied to phMECs within the 3D constructs under physiological (0.3 mm/s) and pathophysiological (0.01 mm/s) perfusion conditions. Therefore disposable flow-through chemo-optic micro-oxygen sensors (PreSens GmbH; Germany) were incorporated into the bioreactor system to obtain online measurements as previously described [16]. Measurements of oxygen levels were acquired every 10 min using Fibox 3 oxygen meters (PreSens GmbH; Germany). Two independent experiments with two bioreactors each were performed (Fig. 1B1).

#### RNA extraction

Engineered constructs were harvested in 1.5 ml tubes, flash frozen on dry ice and RNA extraction was performed subsequently. To this end, frozen constructs were transferred into sterile petri dishes which were placed on dry ice. Constructs were cut into small pieces using a sterile scalpel, transferred into 1.5 ml tubes, and cell lysis buffer added. After vortexing for 30 s, centrifugation was performed at 2000 rpm for 5 min at 4 °C and supernatant was transferred to new 1.5 ml tubes. RNA extraction was performed using the Qiagen RNeasy Plus Mini Kit according to the manufacturer`s protocol. To evaluate the quality of extracted RNA, samples were analyzed using Bioanalyzer Eukaryote Total RNA Nanochip (Agilent) (Fig. 1B2).

#### Library generation and RNA sequencing

Library preparation was performed using TruSeq Stranded mRNA Library Prep Kit (Illumina). In brief, poly-A containing mRNA molecules were purified using poly-T oligo-attached magnetic beads. After purification, fragmentation of mRNA and first and second strand cDNA synthesis was performed. Following ligation of the adapter to the cDNA fragments, products were purified and PCR enriched to create the final cDNA library. The Libraries were sequenced on the NextSeq 500 system SR76 (Illumina). 26–46 million high quality reads were generated per library (Fig. 1B2).

#### Bioinformatic analyses of RNA sequencing data

Fastq files containing reads from MECs cultured under pathophysiological and physiological flow conditions were mapped to the human reference gene expression file (ftp://ftp.ensembl.org/pub/release89/fasta/homo_sapiens/cdna/Homo_sapiens.GRCh38.cdna.all.fa.gz) using Salmon 0.8.2 [17]. To assess differential gene expression, a count matrix generated using R v3.3.1 and tximport v1.2.0 [18], was analyzed using DESeq2 v1.14.1 [19]. For analyzing targeted subsets of genes, as for evaluation of hypoxic response, GO terms for “hypoxia” were obtained from AmiGo [20, 21] and mapped to Ensembl ID using biomaRt [22]. Ingenuity Pathway Analysis (Qiagen) was used to identify biological and molecular networks involved in phMEC response under pathophysiological perfusion conditions.

## Results

### Perfusion bioreactor-based in vitro model of the subarachnoid space

**Fig. 2:**
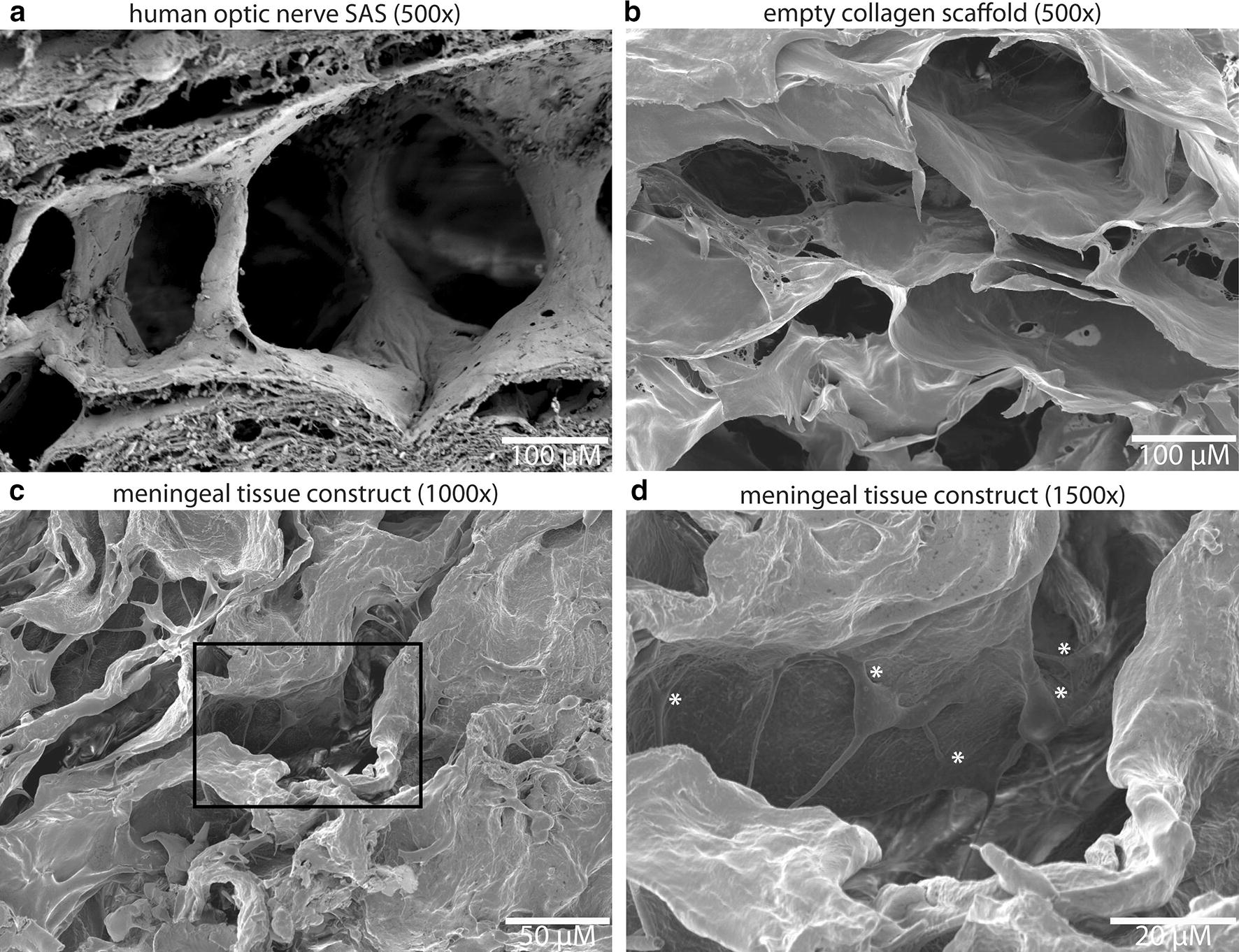
Comparison of microarchitectural similarity between human optic nerve SAS, collagen scaffold and engineered meningeal tissue construct. SEM of human optic nerve SAS (**a**), empty collagen scaffold (**b**) and meningeal tissue construct (**c**, **d**) indicates similarity of the microarchitecture between the 3D in vitro model and in vivo SAS with regards to pore size, geometry and interconnectivity. *Indicates meningothelial cells

phMECs were perfusion seeded into porous 3D collagen scaffolds for 24 h, then cultured for 72 h. After 72 h of culture, phMECs were homogenously distributed throughout the collagen scaffold evident by H&E staining of constructs (Fig. 1A1). Microarchitectural similarity between empty collagen scaffold, engineered meningeal tissue construct and human SAS (optic nerve) was assessed by SEM. Pore sizes of collagen scaffold were in the range of 50–300 μM which was comparable to the pore sizes observed in the meningeal tissue construct and optic nerve SAS. In addition empty collagen scaffolds possessed an interconnected pore structure which was as well present in engineered meningeal tissue constructs and in optic nerve SAS (Fig. 2).

**Fig. 3:**
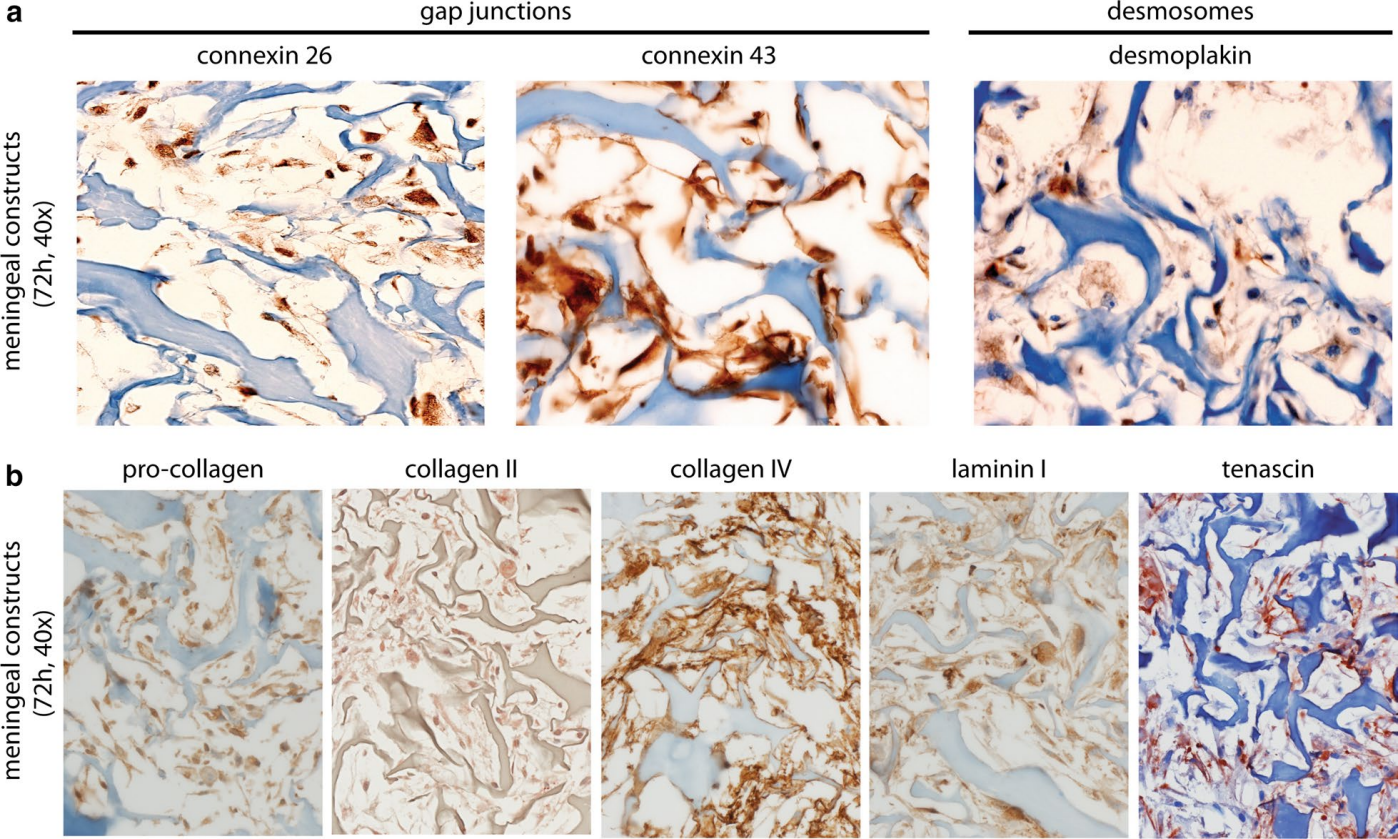
**a** Meningeal tissue constructs revealed immunopositivity for cell–cell interaction markers of gap-junctions (connexin 26 and 43) and weak immunopositivity for desmosomes (desmoplakin) comparable to human optic nerve meninges. **b** Immunohistochemistry to stain for extracellular matrix markers. Meningeal tissue constructs revealed immunopositivity for collagens (pro-collagen, collagen II and IV), laminin I and tenascin. “Blue” indicates structure of collagen scaffold; “Brown” indicates immunopositivity for respective antibody

Engineered meningeal tissue constructs were characterized by staining for cell–cell interaction markers as well as for ECM components, both characteristic hallmarks of native meningeal tissue. After 3 days, phMECs of the tissue-engineered meningeal constructs revealed strong immunopositivity for cell–cell interaction markers of gap junctions (connexin 26 and 43), and weak immunopositivity for desmosomes (desmoplakin) (Fig. 3a). In addition, engineered meningeal tissue constructs showed immunopositivity for ECM proteins pro-collagen, collagen type type II and type IV, laminin and tenascin (Fig. 3b).

### Perfusion bioreactor-based in vitro model of ONCS

#### RNA sequencing

To assess the response of phMECs under physiological and pathophysiological perfusion conditions with RNA sequencing, 6 engineered meningeal tissue constructs were generated under each perfusion condition. After RNA extraction from engineered constructs, Bioanalyzer Eukaryote Total RNA Nanochip (Agilent) was used to determine RNA concentration as well as RNA integrity. Digital electrophoresis revealed no degradation of RNA, evident by the presence of clear bands for 18S as well as 28S rRNA and RIN values ranging from 9.30 to 10.00 (Additional file 1: Figure S1). TruSeq Stranded mRNA Library Prep Kit yielded high quality libraries with library size distribution of an average of 346 bp. RNA sequencing generated 26–46 million high quality reads per library (Additional file 2: Figure S2).

#### Gene expression patterns

**Fig. 4:**
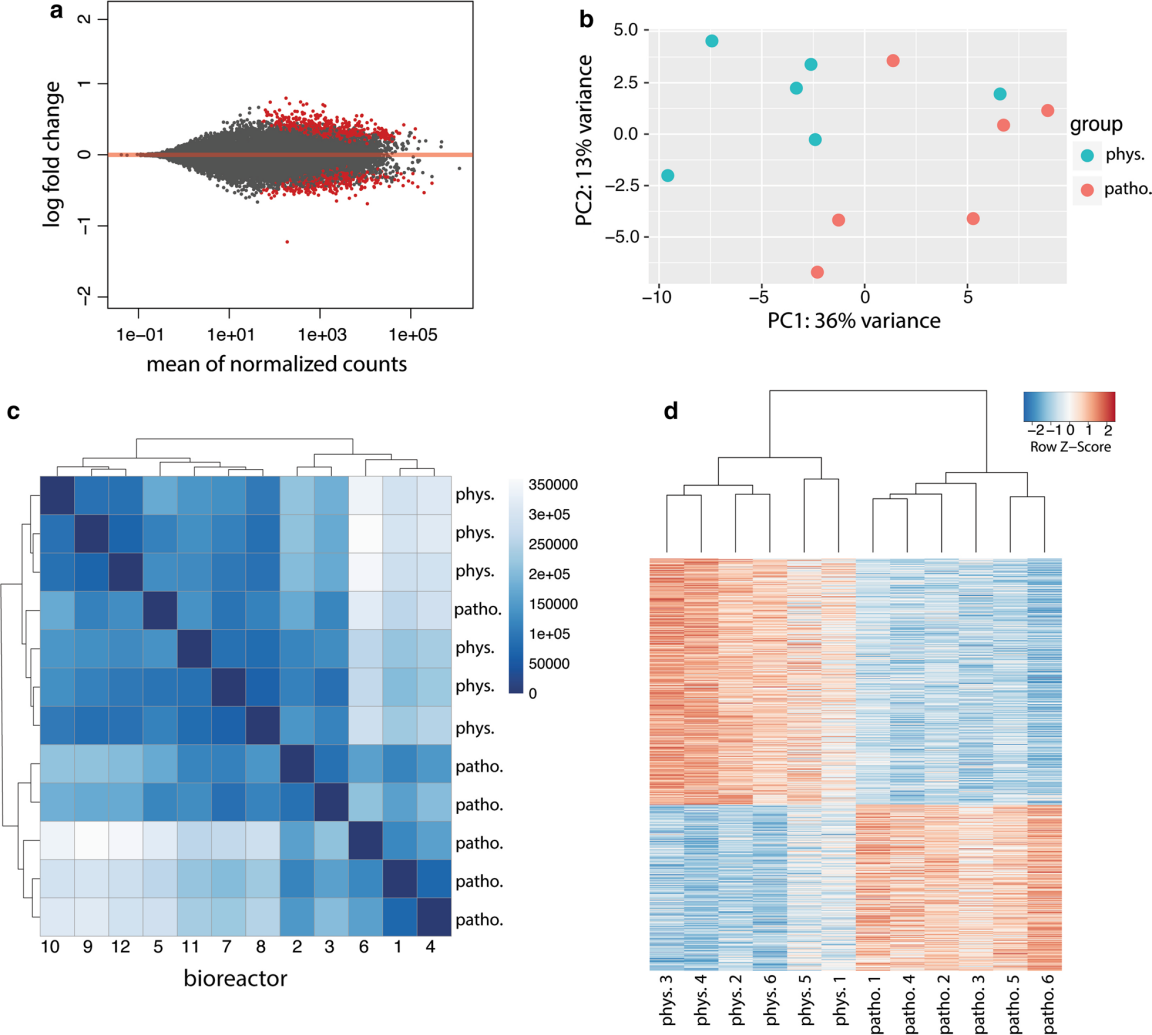
Principal component analysis (PCA) and hierarchical clustering for comparison of gene expression patterns of phMECs under physiological (phys.) and pathophysiological (patho.) flow conditions. **a** MA plot showing DESeq2 filtered genes (25,159 genes). 980 of these genes were significantly regulated (p < 0.1; shown in red). **b** PCA showing clustering of the two treatment groups, respectively. The fraction of the variance is 13% for eigenvector 1 and 36% for eigenvector 2. **c** Hierarchical cluster analysis of the two treatment groups recapitulating observations from PCA. **d** Expression heatmap showing Euclidian sample-to-sample distance matrix with hierarchical clustering of 980 differentially regulated genes in the 2 treatment groups

Following bioinformatics analysis using Salmon for transcript quantification and DESeq2 for differential gene expression analysis, 25,159 genes out of 38,837 genes were found to be expressed in phMECs. Out of these genes, 980 genes were found to be significantly, differentially regulated (threshold = 0) between pathophysiological and physiological perfusion conditions (Fig. 4a). Applying a threshold of 0.5 log2 fold-change, 96 genes were found to be significantly up- or down-regulated. Using principal component analysis (PCA), two main clusters corresponding to the treatment groups could be identified (Fig. 4b. These observations were confirmed in the sample distance analysis (Fig. 4c), with only one sample of the pathophysiological perfusion group clustering with the physiological perfusion group. An expression heatmap comparing Euclidian sample-to-sample distance revealed clustering of pathophysiological and physiological perfusion samples. As shown in Fig. 4d, analysis of differentially regulated genes revealed strong clustering of the two treatment groups.

#### Gene expression patterns of hypoxia related genes

**Fig. 5:**
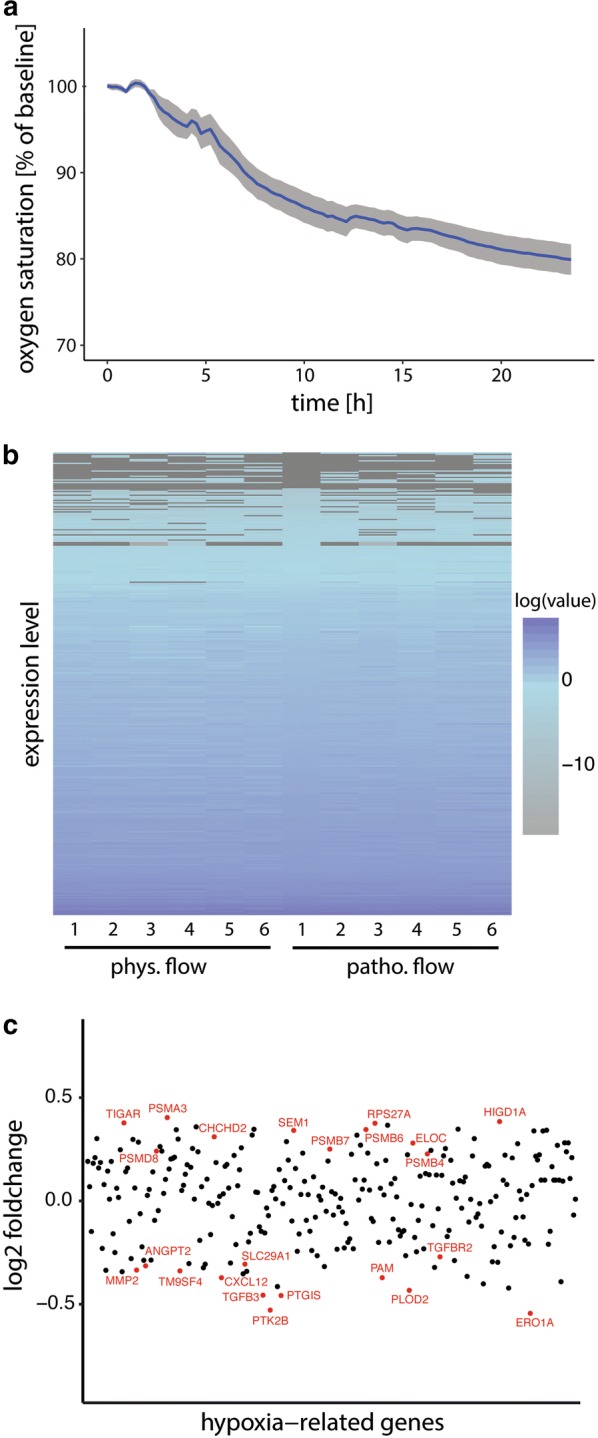
Evaluation of oxygen supply to tissue engineered ONMs and comparison of expression of hypoxia-related genes under physiological (phys.) vs. pathophysiological (pathphys.) perfusion conditions. **a** Oxygen saturation following reduction of perfusion rate to 0.01 mm/s (2.4% of physiological flow, relative to oxygen saturation under physiological flow). Shown is average of two independent experiments with two biological replicates each (S.D. in grey). **b** Expression heatmap showing Euclidian sample-to-sample distance matrix with hierarchical clustering of 362 hypoxia regulated genes in the 2 treatment groups. **c** Blot depicting significantly impacted hypoxia regulated genes

Within the optic nerve, oxygen is supplied to MECs by the vasculature. In our perfusion bioreactor-based model, since oxygen is supplied to cells within the engineered constructs by the perfusion of culture medium, we aimed to verify that the pathophysiological flow rate would not lead to hypoxic conditions in the engineered constructs, which could thereby influence gene expression and RNA sequencing results. Therefore, non-invasive micro-oxygen sensors were integrated into the bioreactor to monitor the level of oxygen supplied to MECs during culture. Figure 5a shows the average relative oxygen profile of two independent experiments with two biological replicates each. During 24 h of culture, only a minor drop in oxygen levels was observed. Oxygen levels in engineered constructs cultured under pathophysiological flow were maintained at 79.8 ± 3.1% compared to levels measured at physiological flow. Based on these results, no hypoxic response would be expected under pathophysiological flow. We next aimed to corroborate the oxygen measurements and analyzed data from RNA sequencing for the expression pattern of 362 hypoxia related genes between physiological and pathophysiological perfusion conditions using hierarchical clustering. No significantly different gene expression patterns for hypoxia related genes between the two treatment groups were observed (Fig. 5b). Evaluation of significantly regulated hypoxia genes revealed both up-and downregulation of a small subset of genes, indicative for lack of specific hypoxic signaling (Fig. 5c).

#### Gene ontology (GO) analyses

**Fig. 6:**
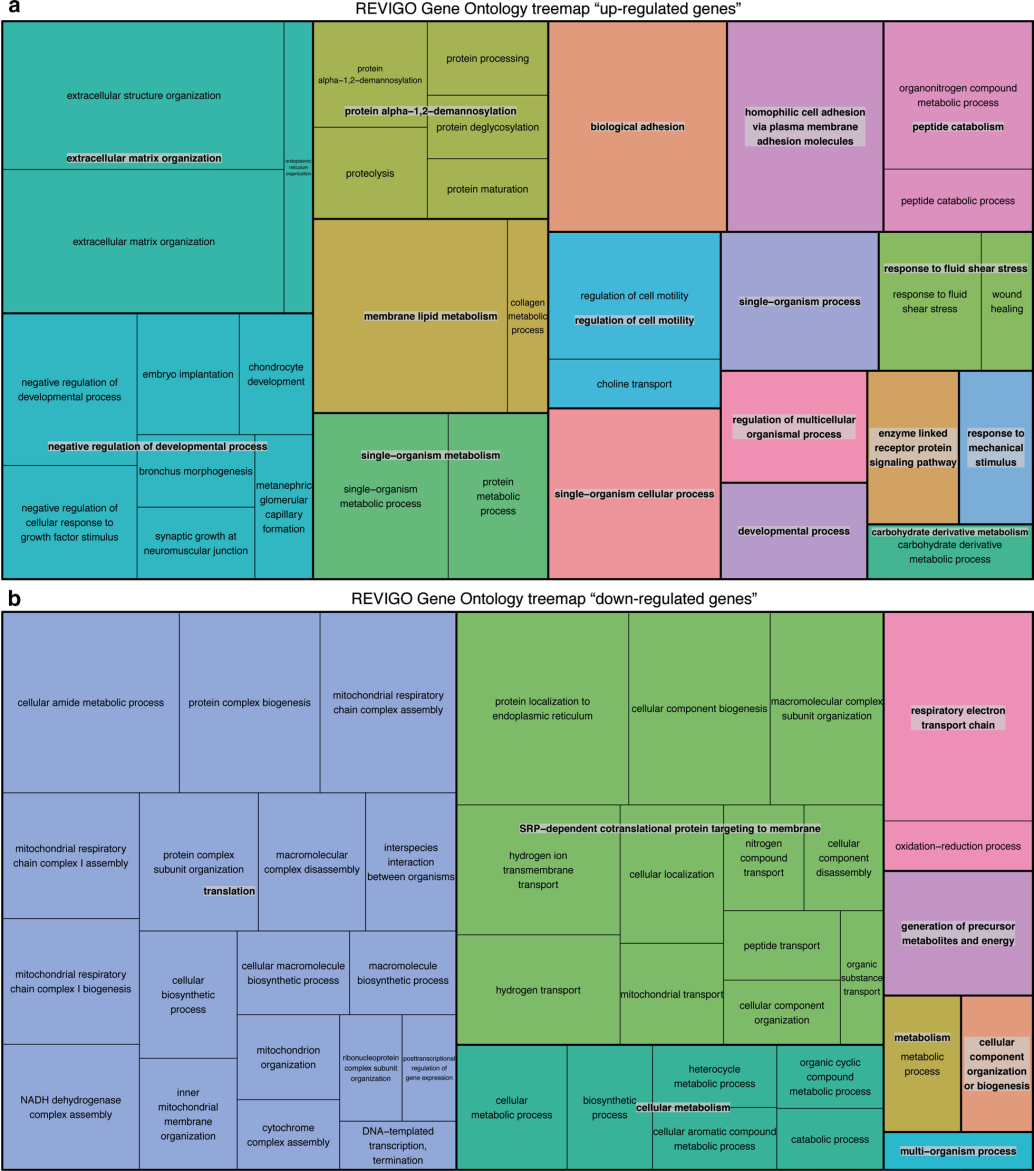
REVIGO analyses of enriched GO-terms for up-regulated and down-regulated genes. **a** Up-regulated genes were found in the categories of extracellular matrix organization, negative regulation of developmental processes, peptide and protein metabolism and cell/biological adhesion. **b** Down-regulated genes were found in the categories of translation, SRP- dependent cotranslational protein targeting to membrane and cellular metabolic processes

Enrichment analysis of gene ontology (GO) terms for the 980 differentially regulated genes revealed several processes, namely mRNA catabolism, cellular and mitochondrial bioenergetics, which were affected by pathophysiological flow (Additional file 3: Figure S3A). These processes were also identified when focusing on GO terms describing biological component (Additional file 3: Figure S3B) or biological function (Additional file 3: Figure S3C). Using REVIGO GO term refinement, significant differentially up-regulated genes were found in the categories of ECM organization, system development, peptide and protein metabolism and cell adhesion (Fig. 6a). Down-regulated genes were found in the categories of translation, signal recognition particle—dependent cotranslational protein targeting to membrane and cellular metabolic processes (Fig. 6b).

#### Pathway analysis

**Fig. 7:**
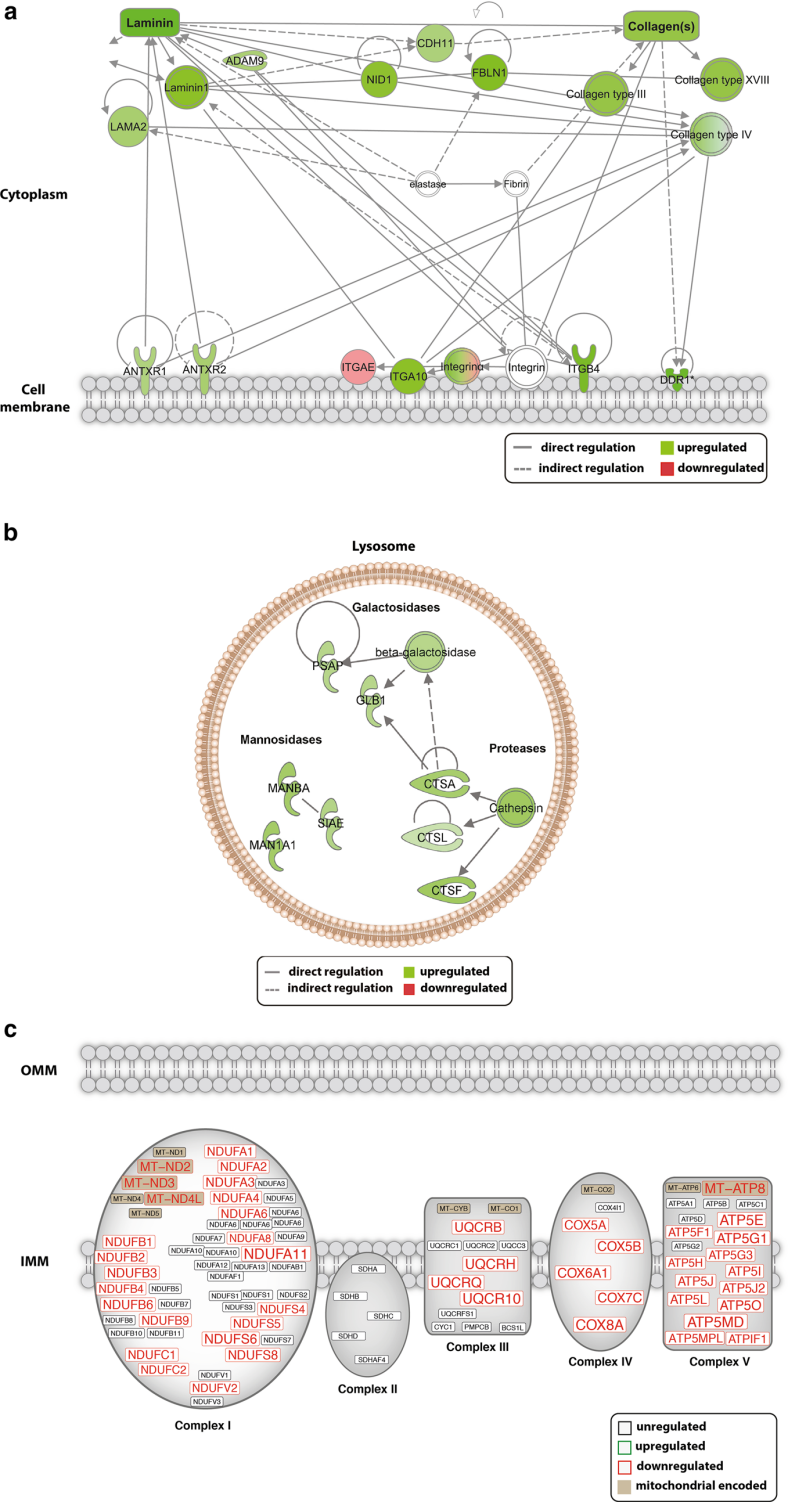
Ingenuity pathway analysis showing regulation of genes that code for components with functions in extracellular matrix remodeling, endo-lysosomal processing and mitochondrial energy metabolism. **a** For extracellular matrix remodeling up-regulated genes belong to members of the laminin (laminin 1/subunits α (LAMB1) and β2 (LAMA2)), collagen (Type III, XVIII, IV) and integrin family (Integrin α). **b** For endo-lysosomal processing up-regulated genes belonged to members of the protease (cathepsins A, L and F), galactosidase (β-galactosidase) and mannosidase (α-mannosidase and β-mannosidase) families. **c** For mitochondrial energy metabolism down-regulated genes found were associated to ETC Complex I, III (ubiquinol binding proteins), IV (cytochrome C oxidase) and V (ATP-snthase) composition and function

To gain further insight into the effect of pathophysiological flow on the regulation of biological networks in phMECs, pathway analysis using Ingenuity Pathway Analysis Software was performed. Consistent with our GO analysis, under pathophysiological flow, pathway analysis revealed an enrichment of up-regulated genes in processes including ECM organisation and endo-lysosomal processes. Genes upregulated in ECM organization included collagens (Type III, IV, XVIII) and laminin components (laminin, LAMB1, LAMA) but also integrins (integrin α) (Fig. 7a). Within the endo-lysosomal pathway upregulated genes belonged to members of the protease (cathepsins A, L and F), galactosidase (β-galactosidase) and mannosidase (α-mannosidase and β-mannosidase) families (Fig. 7b). An enrichment of down-regulated genes was found in the mitochondrial energy metabolic pathway. Within this pathway various genes coding for functional/structural components of the electron transport chain (Complex I, III, IV, V) were found to be down-regulated (Fig. 7c).

## Discussion

We have established an in vitro 3D culture model as a surrogate for the SAS by culturing primary human MECs on collagen scaffolds within a perfusion bioreactor system. This model not only allows us to recapitulate the three-dimensional architecture, the complex cell–cell and cell–matrix interactions but also the fluid dynamics within this microenvironment, thereby making it a representative model to study MEC responses under physiological and pathophysiological conditions.

Studies applying computer assisted cisternography in patients with normal tension glaucoma and papilledema demonstrated impaired cerebrospinal fluid dynamics in the subarachnoid space of the optic nerve, a condition that is now described as optic nerve compartment syndrome (ONCS) [23–26]. Using our established model we demonstrated that pathophysiological flow conditions, as observed during ONCS, altered the trancriptomic profile of phMECs. Transcriptomic changes were observed in processes including extracellular matrix remodeling, endo-lysosomal processing and mitochondrial energy metabolism. We hypothesize that alterations of these processes might not only impact SAS architecture and, thus, flow dynamics but are also likely to interfere with major MEC functions, ultimately impacting optic nerve homeostasis.

The SAS possesses an intricate microarchitecture, being traversed by collagen reinforced trabeculae, pillars and septae which are covered with MECs [27]. Collagen scaffolds have been used for a number of tissue engineering applications. Therefore, to mimic the SAS microarchitecture, biologically as well as structurally, we employed a collagen scaffold which has been shown to possess amorphous interconnected pores [28]. While porous collagen scaffolds have been used for numerous tissue engineering applications, for the first time, we have used a collagen scaffold for the in vitro engineering of meningeal tissue constructs. Scanning electron microscopy showed microarchitectural similarities between the empty collagen scaffold, the engineered meningeal tissue construct with the SAS of a human optic nerve (mid-orbital), with regards to pore size and geometry as well as interconnectivity. Within the SAS MECs form a tightly interconnected cellular network between the CSF and the CNS. While cell–cell interaction proteins are essential for maintaining barrier function and facilitating intercellular communication, meningeal-derived ECM components have been shown to be crucial for basal lamina integrity and thus for neuronal homeostasis. Thus, the presence of cell–cell interaction proteins and ECM components are characteristic features of a functional meningothelial tissue [9]. Characterization of the MEC-based engineered constructs was therefore performed by staining for cell–cell interaction markers as well as ECM components. Immunohistochemistry of our MEC-based constructs demonstrated the presence of cell–cell interaction markers for gap-junctions and desmosomes that have previously been identified within human optic nerve meningeal tissues [29]. In addition, engineered meningeal tissue constructs were consistent with the composition of native tissues [7], staining immunopositive for the ECM components pro-collagen I, collagen II and IV as well as for laminin and tenascin.

Applying our model, we evaluated the response of MECs to conditions of pathophysiological flow dynamics as observed in ONCS and analyzed their transcriptional profile using RNA sequencing. Although there are studies of our and other groups that employ diffusion MRI to measure CSF fluid dynamics within the brain, spinal canal and optic nerve SAS [30, 31], it is at the current time not feasible to obtain absolute CSF flow rates due to technical limitations. However it is possible to measure a so called flow-range ratio that allows the relative measurement of CSF flow rate by diffusion MRI imaging. Flow rates were selected based on diffusion MRI measurements of flow-range ratios between the intracranial cavity and the subarachnoid space of the optic nerves (our unpublished observations). To simulate the significantly reduced pathophysiological CSF flow occurring during ONCS, we used a flowrate in the bioreactor that was dramatically reduced to 2.5% of the normal flow. Although an absolute range of flowrates occurring in ONCS is still to be determined in the literature, the value selected for our model allowed for significantly inhibited flow while at the same time maintaining sufficient mass transport of oxygen to cells in the 3D construct, preventing hypoxic conditions.

RNA sequencing revealed a strong impact of pathophysiological flow on major physiologically relevant MEC functions. Although knowledge about the impact of fluid-induced shear stress on MECs is limited, previous work demonstrated that these cells are highly responsive to changes in fluid dynamics in vivo. Upon experimental blockage of CSF flow in sheep optic nerves, Jaggi et al. observed marked enlargement of MECs and of their intracellular structures [32]. Research on other cell types exposed to fluid-induced shear stress, such as vascular endothelial cells, provide additional evidence that altered fluid dynamics not only has major impact on cell phenotype but also on cell function. Changes in hemodynamics have been shown to initiate mechanosensitive signaling cascades in vascular endothelial cells resulting in modulation of gene expression and alteration of processes related to a variety of cellular functions including cytoskeletal remodeling [33], ECM homeostasis [34] and cellular metabolism [35]. These changes have major influence on vessel anatomy, integrity and function, making vascular endothelial cells a key player in physiological but also pathophysiological vascular remodeling [36, 37]. It is hence conceivable that MECs might in a similar fashion respond to alterations in CSF dynamics.

Indeed, under pathophysiological flow conditions, RNA sequencing, GO-term analyses and REVIGO GO-term refinement revealed enrichment of up-regulated genes in categories related to changes in cellular, biochemical, and biomechanical properties such as ECM remodeling, biological adhesion and response to fluid-induced shear stress. We found upregulation of genes coding for collagens and laminin as well as for integrins. As MECs are known to secret key ECM components of the meninges, alterations in ECM homeostasis might have a severe influence on the microarchitecture of the optic nerve SAS. This might be particularly detrimental in individuals that present with a narrow optical canal. In this context, Wang et al. demonstrated a narrower orbital optic nerve subarachnoid space in normal tension glaucoma patients compared to controls and patients with primary open-angle glaucoma and elevated intraocular pressure [38]. In a study on 56 normal tension glaucoma patients Pircher et al. found a narrower optic canal compared to age-matched non glaucoma controls [39]. In such individuals MEC induced changes of subarachnoid space geometry might easier result in increased CSF flow resistance and finally in optic nerve compartmentation. In addition, as MECs secrete ECM components that contribute to the pial basal lamina, which is in direct contact with radial glial endfeet of the optic nerve fibers, altered ECM homeostasis by MECs would likely have an impact on the neuronal-glial network [40]. Indeed, there is evidence that alterations of ECM composition can lead to loss of basal membrane integrity, thereby interfering with neuronal-glial functioning. Mice that lack basal lamina components show defects in the attachment of radial glial cell processes at the meninges, which in turn has been shown to lead to enhanced neuronal progenitor apoptosis [41].

Disturbed shear stress has also been shown to induce an autophagic response and to modify bioenergetic processes thereby promoting cell survival [42–44]. Under stress conditions, autophagic clearance of damaged cellular organelles and proteins via the lysosomal pathway allow the cell to maintain cellular integrity. Increase of lysosomal components has thus been previously linked to autophagy induction. Wei et al. demonstrated that increased activation of Cathepsin L can stimulate autophagy and antagonize apoptosis in vascular endothelial cells thereby conferring an antiapoptotic effect [45]. In this regard, under pathophysiological perfusion conditions we observed upregulation of genes coding for components involved in endo-lysosomal processing including Proteases (Cathepsins A, L, F), Galactosidases and Mannosidases. It is reasonable to assume that in MECs the activation of an autophagic response might as well be a mechanism to cope with stress conditions induced by disturbed flow dynamics, thereby promoting cell survival. In addition, MECs have been implicated in the clearance of metabolic waste from the CSF and in maintaining CSF homeostasis [9, 10]. As diminished CSF flow might lead to the accumulation of waste products we therefore hypothesize that reduced flow might be an “alarm signal” for MECs to adjust their degradation capacity to cope with an accumulation in waste products. Li et al. showed shear stress lead to the induction of oxidative stress which in turn mediated activation of Jun-N-terminal kinase, thereby initiating autophagy but as well inducing mitochondrial dysfunction due to impaired autophagic flux [35]. Interestingly, under pathophysiological flow conditions we also found down-regulation of genes involved in cellular metabolic processes and particularly in mitochondrial bioenergetics. Specifically, expression of genes involved in the electron transport chain complexes (I, III, IV and V) were impaired.

## Conclusion

We report for the first time, a 3D in vitro model of the subarachnoid space based on an engineered meningeal tissue constructs. This novel bioreactor-based model recapitulates the three-dimensional architecture, complex cellular interactions, as well as fluid dynamics within the subarachnoid space. Applying this model allowed us to investigate fundamental aspects of MEC function under pathophysiological conditions simulating optic nerve compartment syndrome. Future studies based on this 3D model will allow to gain new insights into the role of MECs in the pathogenesis of optic nerve compartment syndrome and associated optic neuropathies and as well will be useful for the in vitro assessment of novel therapeutic treatments.

## Additional files

**Additional file 1: Figure S1.** Agilent Bioanalyzer RNA quality assessment of physiological flow samples (1–6) and pathophysiological flow samples (6–12). A. Digital electrophoresis image depicting degradation profiles of RNA samples. All samples show clear bands for 18S and 28S rRNA indicative of high quality RNA. B. Electropherogram of each sample with corresponding RIN (RIN 9.3–10.0).

**Additional file 2: Figure S2.** TruSeq Stranded mRNA Library Prep quality assessment of physiological flow samples (1–6) and pathophysiological flow samples (6–12). A. Digital electrophoresis image depicting the library size distribution patterns, which reached on average 346.25 bp. B. Electropherogram of each sample showing library size distribution.

**Additional file 3: Figure S3.** Gene ontology (GO) analysis of RNA sequencing. GO analysis of 980 differentially regulated genes sorted according to Process (A), Component (B) and Function (C). q-value indicating GO-term enrichment.

## Data Availability

The datasets used and/or analysed during the current study are available from the corresponding author on reasonable request.

## Abbreviations

CSF: cerebrospinal fluid
ECM: extracellular matrix
MECs: meningothelial cells
ONCS: optic nerve compartment syndrome
phMECs: primary human meningothelial cells
SAS: subarachnoid space
