# Supplementary figures and images for: A perfusion bioreactor-based 3D model of the subarachnoid space based on a meningeal tissue construct
Source: Fluids Barriers CNS. 2019 Jun 13;16:17. doi: 10.1186/s12987-019-0137-6 (PMC6563372; doi:10.1186/s12987-019-0137-6)

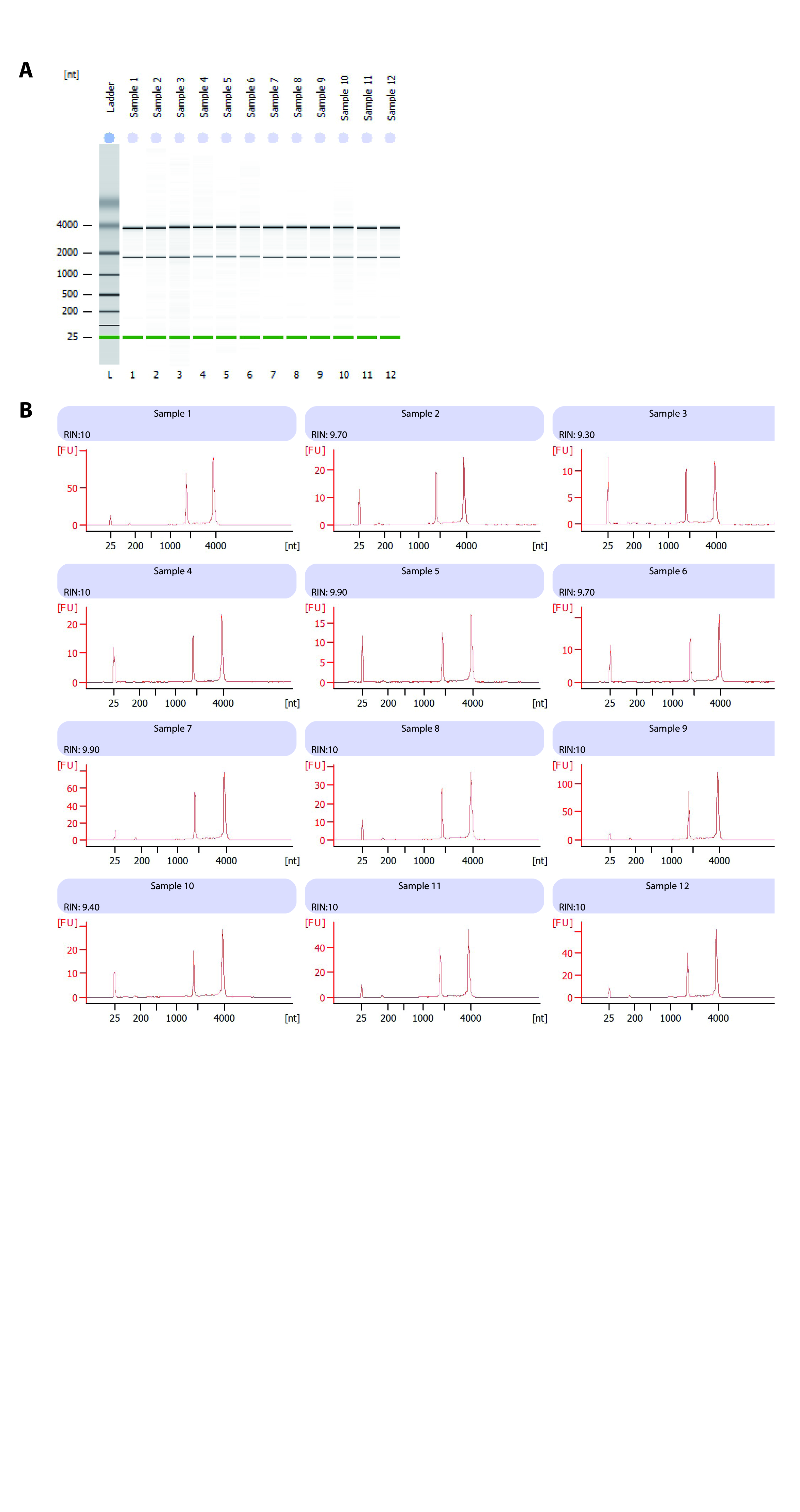

Supplement: Supplementary file 1 — Additional file 1: Figure S1. Agilent Bioanalyzer RNA quality assessment of physiological flow samples (1–6) and pathophysiological flow samples (6–12). A. Digital electrophoresis image depicting degradation profiles of RNA samples. All samples show clear bands for 18S and 28S rRNA indicative of high quality RNA. B. Electropherogram of each sample with corresponding RIN (RIN 9.3–10.0). [file 12987_2019_137_MOESM1_ESM.jpg]

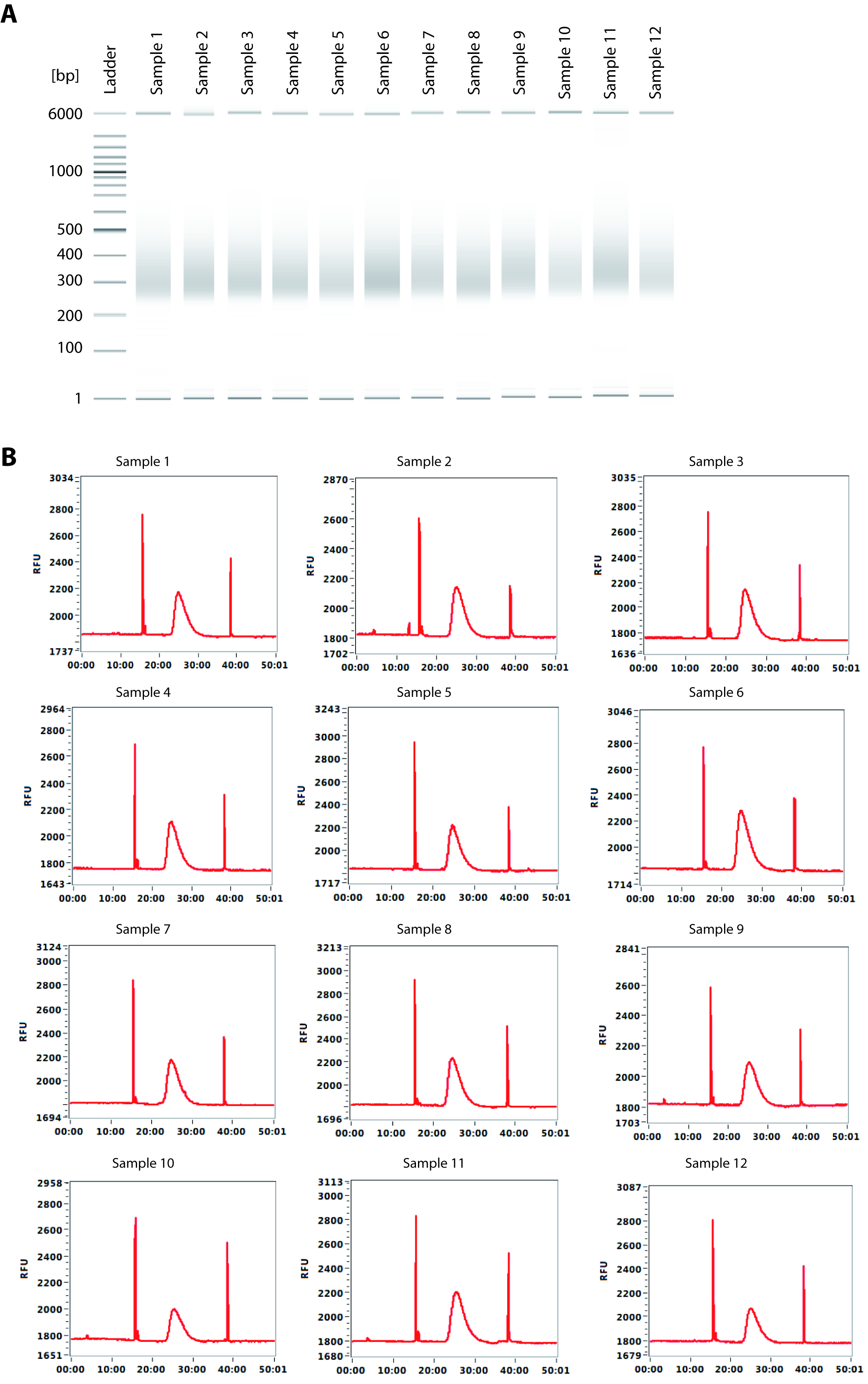

Supplement: Supplementary file 2 — Additional file 2: Figure S2. TruSeq Stranded mRNA Library Prep quality assessment of physiological flow samples (1–6) and pathophysiological flow samples (6–12). A. Digital electrophoresis image depicting the library size distribution patterns, which reached on average 346.25 bp. B. Electropherogram of each sample showing library size distribution. [file 12987_2019_137_MOESM2_ESM.jpg]

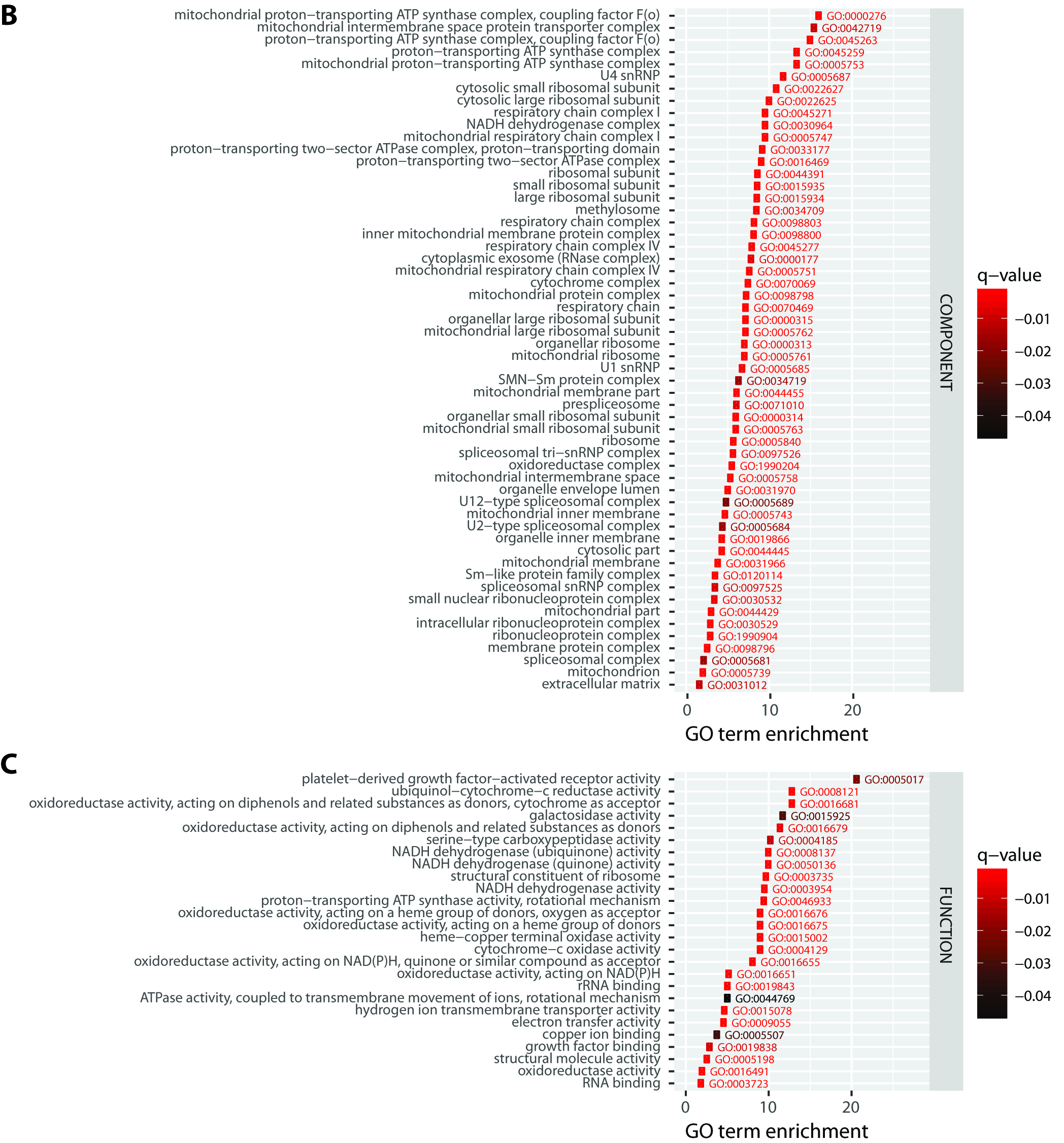

Supplement: Supplementary file 3 — Additional file 3: Figure S3. Gene ontology (GO) analysis of RNA sequencing. GO analysis of 980 differentially regulated genes sorted according to Process (A), Component (B) and Function (C). q-value indicating GO-term enrichment. [file 12987_2019_137_MOESM3_ESM.zip › SupplementaryFigure3_p2.jpg]

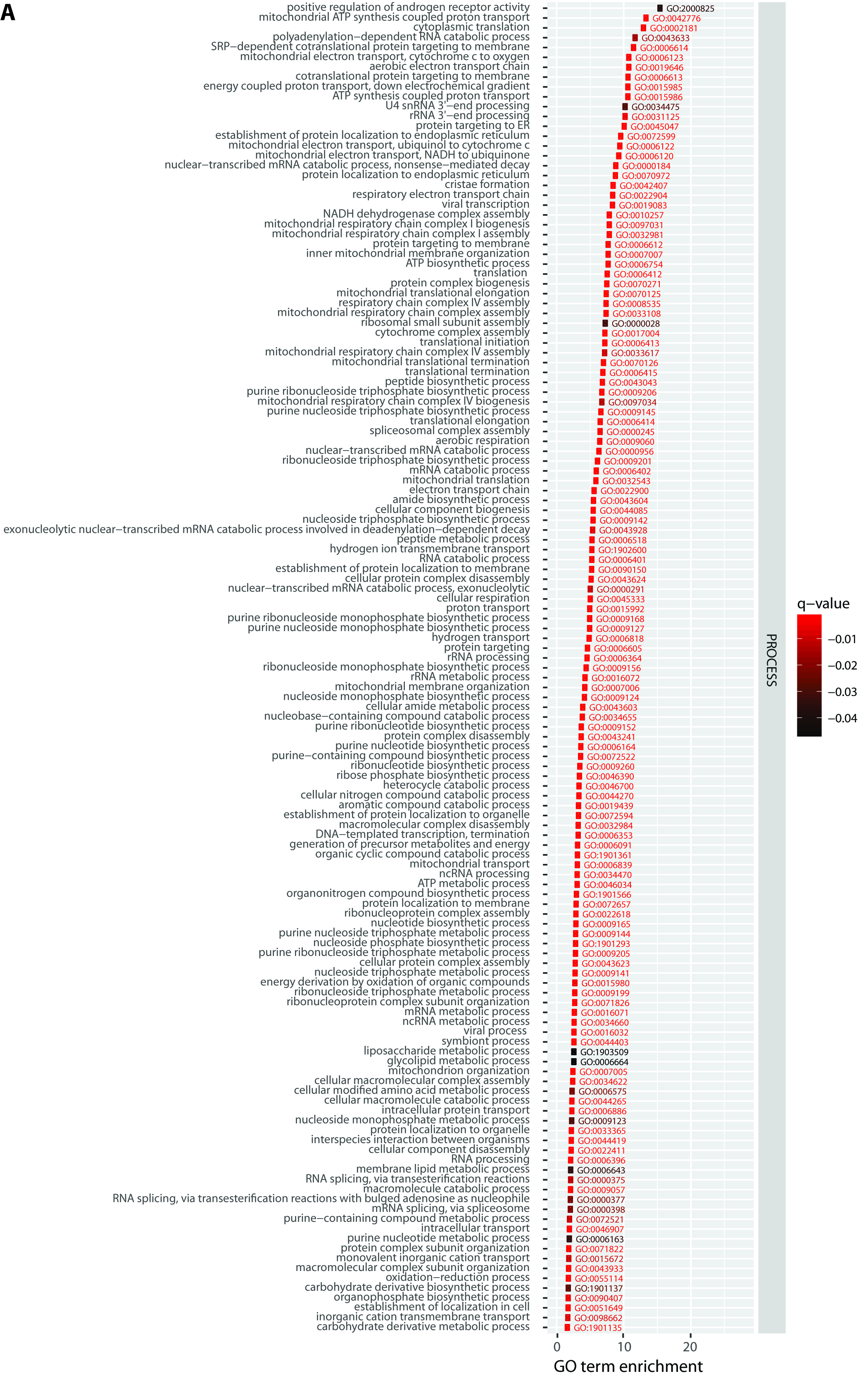

Supplement: Supplementary file 3 — Additional file 3: Figure S3. Gene ontology (GO) analysis of RNA sequencing. GO analysis of 980 differentially regulated genes sorted according to Process (A), Component (B) and Function (C). q-value indicating GO-term enrichment. [file 12987_2019_137_MOESM3_ESM.zip › SupplementaryFigure3_p1.jpg]
